# Synthesis and Investigation of Physicochemical and Biological Properties of Films Containing Encapsulated Propolis in Hyaluronic Matrix

**DOI:** 10.3390/polym15051271

**Published:** 2023-03-02

**Authors:** Gohar Khachatryan, Karen Khachatryan, Magdalena Krystyjan, Lidia Krzemińska-Fiedorowicz, Anna Lenart-Boroń, Anna Białecka, Magdalena Krupka, Marcel Krzan, Karolina Blaszyńska, Monika Hanula, Lesław Juszczak

**Affiliations:** 1 Faculty of Food Technology, University of Agriculture in Krakow, Al. Mickiewicza 21, 31-120 Krakow, Poland; 2 Faculty of Agriculture and Economics, University of Agriculture in Krakow, 30-059 Krakow, Poland; 3 Jan Bober Center for Microbiology and Autovaccines, 31-016 Krakow, Poland; 4 Diagnostyka S.A., (JSC) Medical Microbiological Laboratory, os. Na Skarpie 66, 31-913 Krakow, Poland; 5 Jerzy Haber Institute of Catalysis and Surface Chemistry, Polish Academy of Sciences, 30-239 Krakow, Poland; 6 Faculty of Biotechnology and Horticulture, University of Agriculture in Krakow, Al. Mickiewicza 21, 31-120 Krakow, Poland; 7 Institute of Human Nutrition Sciences, Warsaw University of Life Sciences, Nowoursynowska 159c Street 32, 02-776 Warsaw, Poland

**Keywords:** nanocapsules, antimicrobial properties, *Candida* spp., *Staphylococcus* spp., hyaluronic acid, propolis, nanocomposites

## Abstract

The dynamic development of nanotechnology has enabled the development of innovative and novel techniques for the production and use of nanomaterials. One of them is the use of nanocapsules based on biodegradable biopolymer composites. Closing compounds with antimicrobial activity inside the nanocapsule cause the gradual release of biologically active substances into the environment, and the effect on pathogens is regular, prolonged and targeted. Known and used in medicine for years, propolis, thanks to the synergistic effect of active ingredients, has antimicrobial, anti-inflammatory and antiseptic properties. Biodegradable and flexible biofilms were obtained, the morphology of the composite was determined using scanning electron microscopy (SEM) and particle size was measured by the dynamic light scattering (DLS) method. Antimicrobial properties of biofoils were examined on commensal skin bacteria and pathogenic *Candida* isolates based on the growth inhibition zones. The research confirmed the presence of spherical nanocapsules with sizes in the nano/micrometric scale. The properties of the composites were characterized by infrared (IR) and ultraviolet (UV) spectroscopy. It has been proven that hyaluronic acid is a suitable matrix for the preparation of nanocapsules, as no significant interactions between hyaluronan and the tested compounds have been demonstrated. Color analysis and thermal properties, as well as the thickness and mechanical properties of the obtained films, were determined. Antimicrobial properties of the obtained nanocomposites were strong in relation to all analyzed bacterial and yeast strains isolated from various regions of the human body. These results suggest high potential applicability of the tested biofilms as effective materials for dressings to be applied on infected wounds.

## 1. Introduction

Nanotechnology is one of the most rapidly developing multidisciplinary scientific fields today, drawing on the achievements of chemistry, physics, biology, biotechnology, computer science or mechanics. Nanotechnology allows for the transformation of nanoscience theory into useful and practical applications of nanometric structures [1,2,3,4,5,6]. Thus, it refers to technology that is implemented at the nanoscale and applicable to the real world [7,8,9]. Great advances resulting from the application of nanotechnology and nanomaterials are expected primarily in medicine, biotechnology, agriculture, food technology, information technology, materials and chemical engineering, as well as environmental protection. In recent years, the role of nanotechnology in drug delivery, diagnostics and other related medical fields has increased significantly. Based on their internal structure, polymer nanoparticles can be classified as nanospheres or nanocapsules. Nanocapsules consist of a liquid or solid core surrounded by a polymer coating that allows the encapsulated content to be completely isolated from the environment. This prevents degradation or rupture, which in tissues can be induced by temperature, pH or enzymes [10,11,12,13,14,15]. Above that, the nanocapsule envelopes provide a barrier against factors that can damage the substances encapsulated inside the nanocapsule, such as oxidizing and hydrolyzing compounds [16]. Nanocapsules can be designed to provide a controlled release of active compounds, including encapsulated drugs, giving them a distinct advantage over many injectable drugs and pharmaceuticals [17,18]. The use of nanomedicine advances will allow for the prolonged release and targeted delivery of encapsulated active ingredients [19,20,21,22,23]. The selection of encapsulation technology depends on the required particle size, its physicochemical characteristics, release and delivery methods and the cost of production. There are techniques for which the presence of specialized equipment is required, such as electrospinning, electrospraying and nanospray drying [24,25].

Synthetic polymer composites are gradually being replaced by new ecofriendly materials produced with renewable raw materials. Biocomposites have a number of advantages, such as low production cost, environmental friendliness and biodegradability [26,27,28]. Nanocomposites are materials that have at least one component at the nanoscale, in which the nanofiller and the polymer matrix interact at the molecular level [29,30].

Polysaccharides, natural polymeric materials, are widely used as drug carriers of active compounds due to their biocompatibility, biodegradability, ease and conditions of gelation and established lack of toxicity. Polysaccharides are rich in deprotonated amino groups or carboxylic acid residues, as a result of which the resulting cationic or anionic charges form a polymer coating through electrostatic interactions [31,32].

Hyaluronic acid is a long, unbranched polysaccharide composed of N-acetyl-D-glucosamine and recurring D-glucuron disaccharides. Due to the presence of carboxyl groups, it is a highly hydrophilic compound and capable of forming a viscous network at high molecular weights [2,33]. Hyaluronic acid is present in many organisms, and as much as 50% is found in the dermis and epithelium. There are two main functions of this polysaccharide in the body: as a structural macromolecule and as a signaling molecule. It therefore plays an important role in modulating biological processes, which include signal transduction pathways, angiogenesis or tissue repair processes [21]. Through chemical modifications of hyaluronic acid, it is possible to produce various forms of bioresorbable and biocompatible polymers that can be used in regenerative medicine to produce membranes, gases and gels. Hyaluron has been shown to play a key role in wound healing processes, affecting the proliferative and remodeling phases of the process [33]. In addition, the bacteriostatic properties of this polysaccharide may contribute to the formation of potential therapeutic coatings on wound dressings [4,34,35]. Physiological activity and viscoelastic properties make hyaluronic acid an excellent material for use in pharmacology, especially in ophthalmology, dermatology or rheumatology [29,33].

Bee products have been held in high esteem for centuries, not only for their taste, but also for their unique properties [36,37,38]. Propolis is a natural compound extracted by bees from plant fluids and exudates [39]. From the Greek language, the word “propolis” means defense for the community (“pro”—defensive, “polis”—community), so the main purpose of the preparation of propolis by honey bees is its use in protecting the hive from cracks, smoothing its walls and maintaining a stable temperature and moisture inside [40,41]. The composition of raw propolis consists mainly of resins, waxes and, to a lesser extent, essential oil fractions. Depending on the geographic origin and botanical source, the proportion of these compounds can vary [39,42]. The main components of propolis, which comes from moderate temperature regions, are flavonoids that do not have B-ring substituents, which include chrysin, pinocembrin, galangin and pinocancin. The chemical composition of propolis from tropical regions is dominated by prenylated phenylpropanoids such as artepillin C and diterpenes. On the other hand, propolis from the Pacific and African regions contains geranylflavonones [40]. Propolis also contains many important vitamins, including C, E and B vitamins. In addition, it also contains important minerals such as calcium, magnesium, sodium or potassium [41,43,44]. Propolis has been used in medicine for many centuries due to its known health properties. It exhibits anti-inflammatory, antifungal, antibacterial, antiseptic and anticancer properties. Phenolic and flavonoid compounds, which have high pharmacological and biological activity, were found to be largely responsible for its anti-inflammatory and antimicrobial properties [39,41,45]. However, studies have shown that the observed health-promoting properties of propolis may be the result of the synergistic interaction of more components found in propolis. Above that, propolis shows no toxicity or side effects when used in animal or human models [46,47,48]. The possible antimicrobial mechanism is based on the electrostatic interaction between the cationic groups of propolis extract compounds and the negative charges present on the surface of the cell walls of microorganisms. This type of interaction leads to their damage, and consequently to the elimination of microorganisms [39,49]. The bactericidal nature of propolis can be considered at two main levels. The first is the direct effect of the substances contained in propolis on bacteria, and another is related to the stimulation of the immune system, which results in the activation of the body’s defense mechanisms. Propolis contributes to an increase in the permeability of the cell membrane of microorganisms, interferes with the production of adenosine triphosphate (ATP) and membrane potential, and reduces the motility of pathogens [43,50,51,52].

The nanoencapsulation of propolis extract in matrices of natural, biodegradable polymers would allow for the controlled release of the contents and regular action on pathogens without harming the environment [53,54,55].

The aim of this study was to produce biodegradable polysaccharide biofilms based on hyaluronic acid with nanocapsules from propolis, and to investigate the physicochemical properties of the obtained bionanocomposites. The obtained biodegradable films with encapsulated active compounds were to serve as antimicrobial agents against selected strains of bacteria and yeast.

## 2. Materials and Methods

### 2.1. Materials

Materials included sodium hyaluronate (2.0–2.2 MDa high molecular weight, ALFA SAGITTARIUS, Kraków, Poland); anhydrous glycerin—plasticizer (99.5%, p.a., Pol-Aura, Morąg, Poland); propolis (purified and concentrated ethanolic extract of propolis—P, 90.12% d.m., stored in a dark bottle, in refrigeration conditions (4 °C)) obtained from Laboratorium Bio-Farmaceutyczne (Krakow, Poland); ethyl alcohol from P.P.H. Stanlab Sp. z o.o. (Lublin, Poland); Mueller–Hinton substrate, with bacterial strains identified by the Jagiellonian Innovation Center and yeast strains obtained from CBMiA—for Microbiological Research Center and AutoVaccine (Krakow, Poland); and saline.

### 2.2. Synthesis of Films Containing Encapsulated Propolis in Hyaluronic Matrix

#### 2.2.1. Preparation of Polysaccharide Gels

In order to obtain 2% *w*/*w* hyaluronan gel, a controlled gelation method was carried out, during which 20.0 g of the polysaccharide under study was weighed out and then made up to 1000.0 g with distilled water. The thus-prepared mixture was placed on a magnetic stirrer at 60 °C and allowed to stand overnight until a transparent glue was obtained. After the prescribed time, 10.0 g of glycerin was added to the prepared homogeneous mixture in a ratio of 1:2 relative to hyaluronic acid.

#### 2.2.2. Preparation of Propolis Emulsion

A total of 60.0 g of an emulsion containing propolis was prepared by placing 15.0 g of propolis, 30.0 g of olive oil and 15.0 g of ethyl alcohol in a conical flask (volume 100 mL). The flask was then placed in an ultrasonic bath cooled in an ice bath (temp. 2 °C). The mixture was sonicated at 40 kHz for 25 min to obtain a homogeneous emulsion.

#### 2.2.3. Control Sample Preparation

A total of 20.0 g of distilled water was added to 100.0 g of 2% sodium hyaluronate gel and homogenized for 5 min. A control sample (ControlH) was obtained.

#### 2.2.4. Preparation of Propolis Nanocapsules

To 100.0 g of 2% sodium hyaluronate gel, previously prepared emulsions of propolis (8 g, 12 g and 16 g) were added dropwise, mixing using a homogenizer. Distilled water was then added to the individual samples in amounts of 12.0, 8.0 and 4.0 g, respectively, and further homogenized for 5 min to obtain a homogeneous mixture. Samples designated HP1, HP2 and HP3 were obtained, respectively.

#### 2.2.5. Film Preparation

A total of 30.0 g of gels (individual samples and a control sample) were poured into Petri dishes (90 mm diameter) and placed in an oven at 35 °C until completely dry.

### 2.3. SEM Microscopy

The size and morphology of the produced nanocapsules were analyzed using a JEOL 7550 scanning electron microscope (Akishima, Tokyo, Japan). Prior to the measurements, the samples were sprayed (K575X Turbo Sputter Coater) with 20 nm chromium (Cr) to increase the conductivity of the samples.

### 2.4. UV-Vis Spectroscopy

Spectra were recorded using a scanning spectrophotometer (SHIMADZU TCC-260, Kioto, Japan), in the range of 200–700 nm. Measurements were made for the obtained films (8 × 40 mm^2^ strips) in a quartz cuvette (10 mL, 10 mm thick quartz cells). A blank cuvette was used as reference sample.

### 2.5. FTIR Spectroscopy

Using a MATTSON 3000 FTIR spectrophotometer (Dornstadt, Germany) equipped with a 30SPEC 30° reflectance overlay with a MIRacle ATR accessory, FTIR-ATR spectra of the produced biofilms in the 4000–700 cm^−1^ range were recorded. All spectra were recorded at an instrumental resolution of 4 cm^−1^ using 32 scans.

### 2.6. Surface Color Measurements

The surface color of films was measured using Konica MINOLTA CM-3500d equipment (Konica Minolta Inc., Tokyo, Japan), using a reference D65 illuminant/10° observer, with a 10 mm diameter window. The results were expressed using the CIE L*a*b* system. The parameters, such as L* (L* = 0 black, L* = 100 white); a* (a* < 0 share of the green color, a* > 0 share of the red color); and b* (b* < 0 shares of blue, b* > 0 shares of yellow), were determined on a standard white background.

### 2.7. Thickness Measurement

The thickness of films was measured with a micrometer, catalog no. 805.1301 (Sylvac SA, Crissier, Switzerland), with a 0.001 mm resolution. The sample thickness was the average of five measurements performed in various places within the gauge length area.

### 2.8. Mechanical Properties of Composites

Dry composites were conditioned for 48 h prior to analysis in desiccators at 25 °C and 52% relative humidity (RH) by using saturated solutions of magnesium nitrate-6-hydrate. The samples for textural analysis were prepared according to ISO standards [32]. Mechanical properties of film were carried out using the TA-XT plus texture analyzer (Stable Micro Systems, Haslemere, UK). Strips of films (35 × 6 mm^2^) were prepared and then placed in the holders of the measuring instrument. The initial grip separation between holders was 20 mm and the rate of grip separation was 2 mm/min. Tensile strength (TS) was calculated by dividing tensile force (maximum force at rupture of the film) by the cross-section area of the film. The percentage of elongation at the break (EAB) was calculated by dividing the elongation at rupture by the initial gauge length and multiplying by 100. The final result was the average value obtained from 10 repetitions.

### 2.9. Particle Sizes (DLS) and Zeta Potential

The size and zeta potential of nanoparticles were measured by dynamic light scattering [56] and by laser Doppler velocimetry with Malvern Nano ZS instrument as described earlier [3,12,29]. Samples were diluted with the use of Mili-Q water (resistance over 18.2 MΩ). Each measurement was repeated three times. The Zetasizer enabled the analysis of particles in a size range from ca. 3 nm to 10,000 nm, with an instrument error of the order of 2 nm. The average zeta potential measurement error (standard deviation) was 5 mV maximum. All experiments were performed at room temperature (22 °C).

### 2.10. Contact Angle and Surface Free Energy

Contact angle and surface free energy were measured using the Kruss Drop Shape Analyser DSA100M equipped with a digital camera and environmental cell. The surface free energy was analyzed by the Owens–Wendt method [57,58], as in our previous publications [12,29]. The concept of the analysis is based on the differences in surface free energy, which occur objectively and regardless of the precision and the performing conditions of the contact angle measurements of two liquids, water and diiodomethane. This methodology is generally accepted as the best for polymer substance evaluations. The test chamber temperature was controlled using a thermostatic water bath allowing for constant temperature (22 ± 0.3 °C) and humidity. For each sample, at least five successive measurements were carried out.

### 2.11. Moisture Content and Moisture Uptake

#### 2.11.1. Water Content

The composites were cut into a rectangle specimen (2 × 2 cm^2^) and weighed in analytical balance, obtaining initial weight of sample (W1). Samples were stored in saturated magnesium chloride solution at 25 °C and 32.8 RH for 24 h. Then, specimens were dried at 70 °C in an oven for 24 h, and the initial dry mass (W2) was then analyzed gravimetrically.

water content (%) = (W1 − W2)/W1 × 100 (1)

where:

W1 is the initial weight of the composites;

W2 is the weight of the composites after drying.

#### 2.11.2. Moisture Uptake

Since composites are very sensitive to water and can partially dissolve in a short time of exposure to water, the method of moisture uptake described by Tajik et al. [59], with some modification, was used. The samples were weighed (W1) and stored in saturated magnesium chloride solution (as in the water content method). Then, specimens were moved to desiccators and kept at 25 °C and 75.3% relative humidity (RH) by using saturated solutions of sodium chloride for 24 h. Afterwards, the specimens were weighed (M3). The moisture uptake values of the samples were calculated using the following equation:

% moisture uptake = (W3 − W1)/W3 × 100 (2)

where:

W1 is the initial weight of the composites;

W3 is the weight of the composites after exposure to 75.3% RH for 24 h.

Each test consisted of triplicate measurements and was expressed as the mean value.

### 2.12. Differential Scanning Calorimetry (DSC)

DSC analysis of the films with propolis was performed using the differential scanning calorimeter DSC 204f1 Phoenix (Netzsch, Germany) according to Pająk et al. [60]. Film samples (approx. 1 mg) were closed hermetically in aluminum pans and heated from 30 to 300 °C at a rate of 10 °C/min. An empty aluminum pan was used as a reference. Temperatures and enthalpy of thermal transitions were determined with the use of the instrument’s software Proteus Analysis (Netzsch, Germany). Onset (To), peak (Tp), (Te) end (Tg) temperatures, and enthalpy (ΔH) of thermal transitions were determined. Each sample was measured in triplicate.

### 2.13. Microbiological Analyses

#### 2.13.1. Isolation and Identification of Microorganisms

Swab samples were collected from various regions of human skin and body, i.e., various types of skin lesions, under-eye region, cheeks, hands, back, eye, ear, mouth, throat, tonsils, vagina, anus. Isolation and identification of bacterial and yeast strains were conducted as described in Khachatryan et al. [35]. Briefly, the samples were inoculated on general and selective media for the isolation of bacterial and yeast strains. The systematic position of bacterial strains (*n* = 8) was determined by MALDI-TOF (matrix-assisted laser desorption/ionization time of flight) mass spectrometry, while the systematic position of yeasts (*n* = 18) was determined with the CandiFast test kit (ELITechGroup, Puteaux, France).

#### 2.13.2. Antimicrobial Activity of Films Containing Encapsulated Propolis

Antimicrobial activity of films containing encapsulated propolis in hyaluronic matrix was assessed on a total of 26 microbial strains. Each of eight commensal bacterial isolates (all originating from the human skin) belonged to different species, i.e., *Microsoccus luteus*, *Staphylococcus aureus*, *S. cohnii*, *S. epidermidis*, *S. haemolyticus*, *S. pasteuri*, *S. saprophyticus* and *S. warneri*. Eighteen pathogenic *Candida* isolates (two type strains and sixteen isolates from various regions of human body) belonged to seven species: *C. albicans*, *C. formata*, *C. glabrata*, *C. guillermondi*, *C. inconspicua*, *C. krusei* and *C. parapsilosis* (Table 1). Apart from the type strains, Candida spp. were isolated from swabs from anus, vagina, cheek, mouth, under eye, skin and throat.

Microorganisms were transferred to sterile saline solution to obtain 0.5 MacFarland suspensions and they were streaked onto Mueller–Hinton agar (Biomaxima, Lublin, Poland). The films containing encapsulated propolis were sterilized under UV light for 30 min. Then, 10 × 10 mm^2^ squares were cut with sterile scissors and applied onto the surface of microbial cultures with a sterile tweezers. The cultures were incubated at 35 °C for 18–24 h (in case of bacteria) and for 3–5 days (in case of yeasts). After the time of incubation, the results were read by observing whether the growth of microorganisms was inhibited. The growth inhibition zone diameters were measured around the film fragments. Due to the fact that the applied films were square, two diameters were read, and the final result was expressed as a mean of the two reads (mm). All experiments were performed in three replications.

### 2.14. Statistical Analysis

Statistical analysis was performed using Statistica version 13.3 software (StatSoft, Tulsa, OK, USA) The one-way analysis of variance (ANOVA), Tukey HSD test (confidence level 0.95, significance level 0.05) was carried out.

## 3. Results and Discussion

### 3.1. Scanning Electronm Microscopy (SEM)

**Figure 1:**
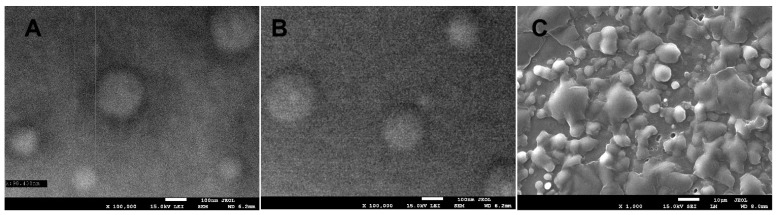
SEM images of a hyaluronic bionanocomposite containing nanocapsules with propolis: (**A**)—HP1 at 100,000× magnification; (**B**)—HP2 at 100,000× magnification; (**C**)—HP3 at 1000× magnification.

SEM images confirmed the receipt of nanocapsules in the sodium hyaluronate matrix (Figure 1). The results show that the nanocapsules obtained in samples HP1 and HP2 have a spherical shape, the size ranges from 90 to 200 nm. The influence of the concentration of the added nanoemulsion on the size and formation of nanocapsules was also observed. The most homogeneous in size nanocapsules were formed in the HP2 sample, while the higher concentration of the nanoemulsion in the HP3 sample resulted in partial encapsulation, and the presence of oil and non-encapsulated extract made it impossible to take pictures at higher magnifications.

### 3.2. UV-Vis Spectroscopy

**Figure 2:**
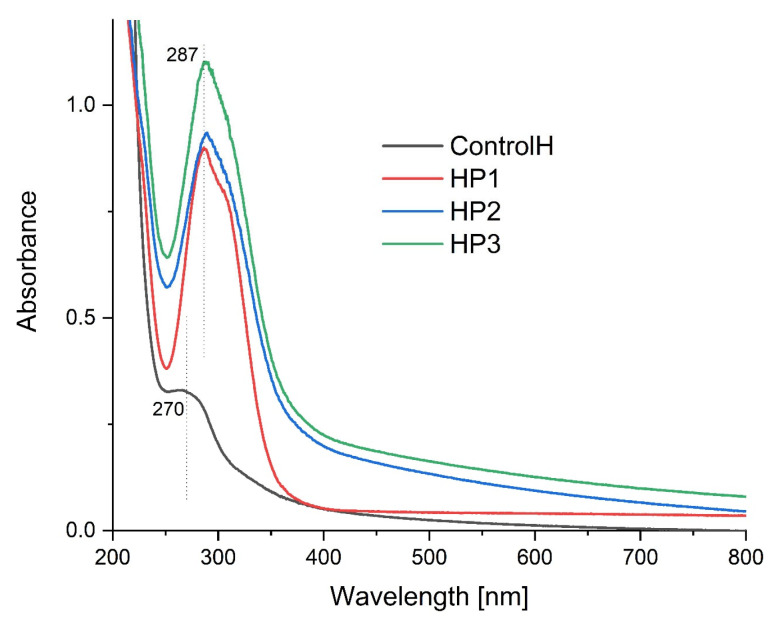
UV-Vis spectrum for the obtained films.

Figure 2 shows the ultraviolet and visible absorption spectra of the obtained films. The addition of emulsion with propolis causes an increase in absorbance in the entire tested range, especially for the HP2 and HP3 samples. For all samples, we observe a peak absorbance in the range from 250 to 380 nm. The HP3 film has the highest absorbance. The presence of phenolic acids and their derivatives, such as flavones, flavonols, flavanones and flavonoids, are revealed by peaks in the UV area between 250 and 400 nm. The propolis sample’s absorbance around 280 nm and at 320–330 nm suggests that it may contain chemicals from the flavanol class [61,62]. Other propolis bands (at lower wavelengths attributed mainly to the aliphatic dienes and carboxylic acids) coincide with bands from sodium hyaluronate and olive oil. There is a difference in absorbance between the samples containing propolis due to the difference in concentration and degree of encapsulation. As there was no change in the absorption maxima, it indicates that the prepared nanoparticles still contain the same bioactive compound [63,64].

### 3.3. ATR-FTIR Spectroscopy

**Figure 3:**
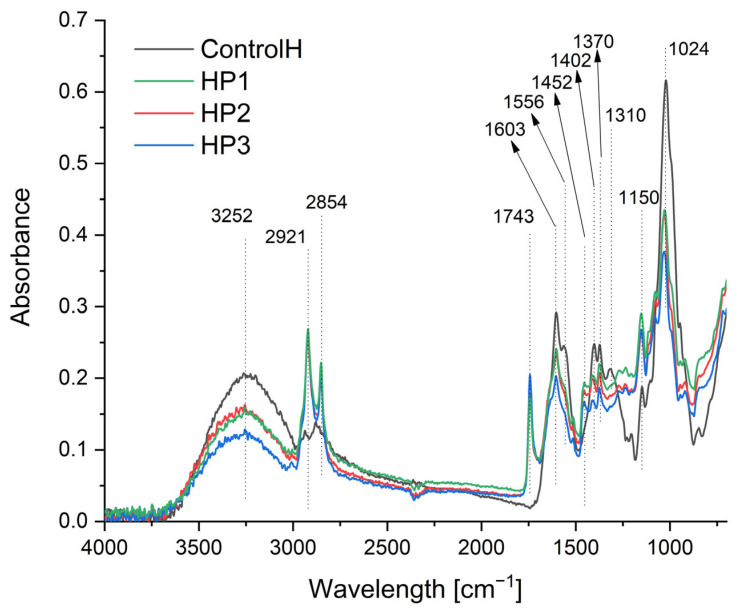
FTIR spectra of the obtained films.

The ATR-FTIR spectra in the spectral range of 700–4000 cm^−1^ for ControlH and nanocapsule coaching films are presented in Figure 3. The FTIR spectrum of ControlH shows the characteristic bands for Hyalironian. The peak located at 3252 cm^−1^ is associated with the intra-and intermolecular stretching vibration of the –OH group and the stretching vibration of the hydrogen bond from the –NH– group. Stretching vibrations of the methylene (–CH_2_–) and methyl (–CH_3_) groups can be observed at the frequencies of 2921 and 2854 cm^−1^, respectively. The band at about 1603 cm^−1^ corresponds to the amide carbonyl and the band at 1402 cm^−1^ can be attributed to the stretching of the COO– group; the peak from 1024 cm^−1^ is attributed to the linkage stretching of C–OH [65,66,67]. In the spectra of the obtained samples HP1, HP2 and HP3, we can observe a change in intensity for bands in the range from 2700 to 3500 cm^−1^, resulting from lower water content (stretching vibration of the –OH group) and additional aliphatic groups from olive oil and propolis components (stretching vibrations-C-H). We also observe a characteristic intense absorption band at 1743 cm^−1^ for the ester bond (C=O stretching vibrations) and C–H deformations and aromatic stretching at 1452 cm^−1^ assigned to flavonoids (hydrocarbons (–CH_3_ and –CH_2_– vibrations are overlapping) [66,68]. Analyzing the obtained results, it can be concluded that hyaluronic acid is a good matrix for the preparation of nanocapsules, because there are no chemical reactions between the matrix and nanocapsules, and the structure of the biopolymer remains intact. In the spectra of individual samples, there are slight differences in absorbance intensity, which may be due to the difference in concentrations of propolis and olive oil. Interactions of the type of van der Waals interactions, the formation of hydrogen bonds between the emulsion components (propolis) [40] and the polysaccharide are most likely to occur, thanks to which, among others, the resulting capsules are stable.

### 3.4. Surface Color Measurements

The color parameters (L*, a*, b*) were determined, which are presented in Table 2. The film containing the lowest propolis concentration had the lowest brightness (87.8) and predominantly green and yellow tones (−3.7 and 30.4, respectively). As the propolis concentration increases, the proportion of red and blue increases. This is a consequence of the presence of colorful substances in propolis [69,70]. A similar trend of change in the saturation of the a* component in their study involving propolis was observed by Siripatrawan et al. [71]. Additionally, it was observed that the brightness parameter (L*) ranged from 87.8 to 91.1 in the films with propolis, indicating that the films were bright. Nevertheless, the analysis of the L* color components showed that the brightness parameter increased with increasing propolis concentration in the film. This result may be due to the obtained nanocapsule sizes, which ranged from 1–100 µm; moreover, an increase in the concentration of the substance constituting the core of the capsule contributes to an increase in the formation of the number of capsules (characterized by smaller nanocapsule sizes), and thus the formation of a more extended, stable structure. The color of the film may affect the acceptability of the products when a light-colored food film is used [71].

### 3.5. Mechanical Properties of Composites

The tensile strength (TS) and the percentage of elongation at break (E) of hialuron-based films with the addition of propolis are presented in Table 3. There were visible effects of the propolis addition on hialuron-based films, and this was confirmed by statistical analysis. The addition of propolis caused a decrease in the TS parameter in comparison with control sample. The results indicate that the obtained films are mechanically stronger than the commonly used LDPE films (11 and 37.9 MPa) [72], whereas there were no changes in the elongation at break between samples.

Based on our previous studies, it appears that the mechanical properties of hyaluronan-based films as a carrier depend on the type of agent introduced into the matrix [29]. With the incorporation of silver nanoparticles, the structure of the nanocomposite was strengthened and its stiffness increased. On the other hand, the appearance of an additional polymer in the matrix—lecithin—worsened the mechanical parameters of the film, even compared to the control (hyaluronan matrix). In the opinion of many authors, the method of synthesis and the presence of other substances accompanying the polymer affect the rigidity of the composites [21,29,73]. In the conducted experiments, the stretchability of the film was at a fairly low level (about 4%) (Table 3). However, other studies also confirm the low extensibility of hyaluronan-based composites [74]. Mechanical attributes of nanocomposites are crucial for the use of materials in a wide variety of medical applications [75]. Although the results show deficient mechanical properties, especially extensibility, hyaluronan biopolymers may still have an important function in terms of their potential application in medicine and other industries, where this parameter is not a major disadvantage [21].

### 3.6. Particle Sizes (DLS), Zeta Potential, Contact Angle and Surface Free Energy

As can be seen, the samples have almost the same dispersive properties independent of the composition (amount of additives). On the contrary, samples’ polar energy (and hydrophobic properties) varied from almost perfectly hydrophilic with minimum polar energy (HP3) to nearly perfectly hydrophobic (HP1) (Table 4). The zeta potential and particle size also varied with the propolis concentration in the sample. Analyzing all the values and magnitudes of changes in the surface free energy, zeta potential and aggregate sizes, one can conclude that we reach some optimal content with the increase in the propolis content, at which we observe the best parameters of all the properties mentioned above. Exceeding this critical concentration causes the reversal of the trend and the destruction of the optimal system. The optimal arrangement is sample HP1, fully hydrophilic, with the highest measured polar energy and total surface free energy, in which aggregate sizes of 8000 nm are observed.

DLS is unsuited for mixed samples, and unstable samples produce incorrect findings when using the light-scattering technique. With smaller nanoparticles, it likewise appears to provide less favorable outcomes. However, the technique does provide information about the dynamics that microscopic techniques cannot [76]. The diameter obtained from DLS analysis comprises the particle itself, as well as the ionic interaction between nanocapsules, the polysaccharide chain and the water molecule layer surrounding the NPs, which causes a difference between the particle size analysis performed by SEM and DLS. The water molecule-containing solvation layer surrounds the particle, giving it a larger diameter than it actually is [77]. A discrepancy in size results was also observed by Rodrigues et al. [78] in β-carotene/protein-based capsules, additionally explaining the swelling of capsules in an aqueous environment.

### 3.7. Moisture Content and Moisture Uptake

Due to the main ingredient, sodium hyaluronate, the resulting films are very sensitive to water. Attempts to carry out water absorption using classical methods turned out to be ineffective. Even after a short contact with water, the films decomposed and formed gels and emulsions (in the case of films containing encapsulated oil and propolis). Similar results were obtained by the authors examining composites based on dissolvable hyaluronic combined with bacterial nanocellulose [79], soluble soybean polysaccharide [79] and cellulose within corn starch and chemically modified starch microparticles [80].

The resulting films were characterized in terms of water content and moisture absorption capacity. The results are shown in Table 5. The obtained results correlate with contact angle values and mechanical properties. The ControlH and HP2 samples have comparable values of water content and water absorption capacity, while the lowest moisture uptake value is characterized by the HP3 sample, which has the highest value of contact angle with water, which results from high hydrophobicity.

### 3.8. Differential Scanning Calorimetry

**Figure 4:**
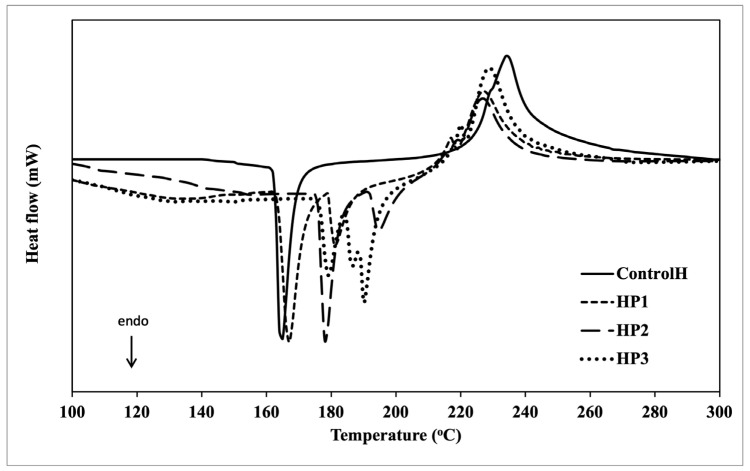
DSC thermograms of hyaluronic films with propolis.

The results of the DSC measurements of composite hyaluronan film with propolis are shown in Table 6 and in the thermogram in Figure 4. The analysis shows that with the increasing propolis concentration in the film, T_o_ increases in the range from 165.5 °C (control) to 183.3 °C (HP3). The data obtained indicate that the addition of propolis increases the thermal stability of the film, probably due to an increase in structural stability due to the encapsulation process. This is in contrast to the results obtained by Pająk et al. [60], in which the addition of propolis in the starch film reduced T_m_, probably due to the less stable crystalline structure of the film. DSC thermograms revealed that the control film showed only one endothermic process and one exothermic process, whereas two endothermic processes and two exothermic processes were observed in the films with propolis addition, which are due to the presence of additional substances contained in propolis (Figure 4). Suriyatem et al. [81] found that propolis can decompose at >150 °C. Their analyses show that the melting point of propolis ranges from 180 °C to 201 °C depending on the type of film tested; the increase in melting point is probably related to the protective role played by the capsules. The analysis of the enthalpy of melting (H) decreases as the proportion of propolis in the film increases (188.7—control, 148.2—HP3). When analyzing the exothermic process of propolis-infused films, depending on the concentration of propolis used, T_o_ increases with increasing propolis concentration; however, the highest T_o_ was observed in the control variant. A similar trend is observed in the analyzed parameter ΔH, which ranges from 197 (HP1, HP2) to 220 (control). Furthermore, according to Villalobos et al. [82], an increase in the T_g_ of semicrystalline and/or amorphous material reveals an increase in the intermolecular forces between the polymer chains, decreasing the local elasticity of the chain by increasing the T_g_, decreasing the chain rotation capacity and giving more rigidity to the film. In the propolis films analyzed, we observe an increase in the T_g_ parameter during the endothermic process, indicating a reduction in elasticity compared to the hyaluronan films.

### 3.9. Microbiological Analyses

**Figure 5:**
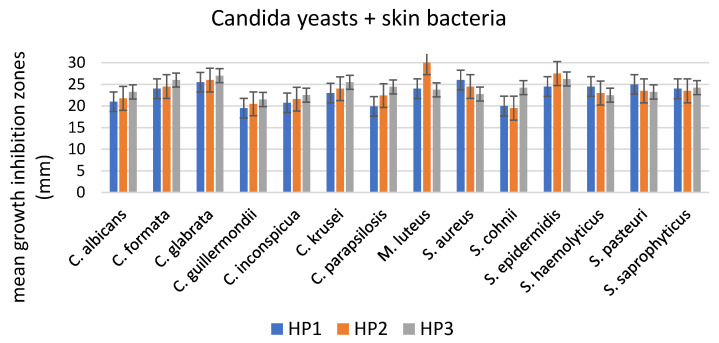
Mean growth inhibition zones (mm) caused by films containing three concentrations of propolis. The results are means of three replicates for the examined isolates of pathogenic Candida (n = 18) and commensal skin bacteria (n = 8). Error bars represent standard deviations.

**Figure 6:**
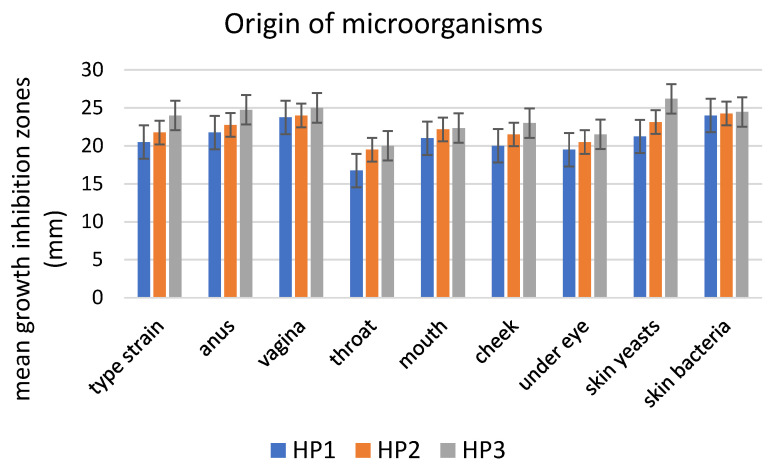
Mean growth inhibition zones (mm) caused by films containing three concentrations of propolis against microorganisms (*Candida* spp., n = 18 and commensal skin bacteria, n = 8) depending on the region of the human body the microbial isolates were obtained from. Error bars represent standard deviations.

The growth of all microbial isolates was inhibited as a result of the application of films containing encapsulated propolis (Table 1, Figure 5 and Figure 6). The growth inhibition zones varied depending on the species of microorganisms, and generally increased with the concentration of propolis in the films. Statistical analysis showed that the differences in the strength of antibacterial effect did not differ significantly between various concentrations of propolis in the films (*p* < 0.05). In the case of the *Candida* yeasts, the antifungal effect of HP3 differed significantly from the HP1 concentration (Tukey’s statistic value of 0.0008, *p* < 0.05). In the case of various species of microorganisms, the difference in growth inhibition of only *M. luteus* and the remaining species was statistically significant (Tukey’s statistic value from 0.026 for *M. luteus* vs. *S. cohnii* to 0.049 for *M. luteus* vs. *C. guillermondii*). In terms of the origin of the microbial isolates, the only statistically significant difference was observed between the isolates originating from throat swabs and the ones obtained from other regions of the human body (Tukey’s statistic values were as follows: for HP1 throat vs. anus 0.025; throat vs. mouth 0.035; throat vs. vagina 0.0009; throat vs. skin 0.0007; for HP2 throat vs. vagina 0.039; throat vs. skin 0.023; for HP3 throat vs. anus 0.030; throat vs. vagina 0.010; throat vs. skin 0.005).

Incidence of fungal infections in humans has increased significantly over the last few decades and the increased and widespread use of immunosuppressive therapies, invasive surgical procedures and broad-spectrum antimicrobial agents are among the most common reasons for such situations [83]. The members of the genus *Candida* are the most frequently isolated from fungal infections of humans. The success of these commensal microorganisms as opportunistic pathogens is due to different virulence factors contributing to their pathogenesis, capacity for adapting to fluctuations in environmental pH, metabolic flexibility, strong nutrient acquisition systems and effective stress response machinery [84]. Due to the occurrence of *Candida* strains resistant to antifungal agents and the fact that the range of drugs available for the treatment of fungal infections is limited to polyenic and azolic compounds [85], the use of natural products is currently being discussed as an alternative to the conventional treatment procedures [86]. An evident growth inhibition of all *Candida* spp. isolates has been observed along a clear increase in the antifungal properties in films containing higher concentrations of propolis (Table 1, Figure 5 and Figure 6). Similar to in the study by [85], a very small variation (statistically insignificant) was observed between the susceptibility of different species of pathogenic *Candida* to propolis containing films. The activity of propolis extracts against Candida yeasts has been reported by Rivera-Yañez et al. [84], Dota et al. [85], Moghim et al. [87], Stähli et al. [86] and Shehu et al. [88]. A detailed chemical composition analysis by Rivera-Yañez et al. [84] allowed the components of propolis with most profound antifungal properties to be distinguished. Among these, various flavonoids, such as pinocembrin, naringin and naringenin, are capable of recognizing and binding to enzymes such as topoisomerase II in *Candida* spp. [89]. Other antifungal compounds included baicalein, capable of inhibiting biofilm formation; and rhamnetin, which has been shown to have anti-*Candida* effects but whose detailed mechanisms have not yet been elucidated [84].

With a variety of chemical compounds, among which polyphenols and terpenoids are considered to be the most active antimicrobial agents, the antimicrobial activity of propolis also includes its antibacterial affect [43]. The antibacterial impact results from its effect on the permeability of bacterial cellular membranes and disruption of their potential and ATP production, as well as decreasing bacterial motility [43]. This, coupled with inducing the activity of the human body’s immune system, makes propolis a bee product of significant importance. In our study, the growth inhibition of skin-derived bacteria, including those of the genus *Staphylococcus*, has also been observed, and only minor differences in the susceptibility of bacteria to this product have been observed as compared to the pathogenic *Candida* isolates. The effectiveness of propolis extracts against both Gram-positive (e.g., *S. aureus*) and Gram-negative (*E. coli*) bacteria has been observed by de Campos et al. [39] and Stähli et al. [86], and summarized by Przybyłek and Karpiński [43].

Due to numerous health benefits from using propolis, the extracts of this compound have been recommended as agents effective in damaged tissue reconstruction, wound healing and treatment of candidiasis, as well as in treatment of burns and frostbite or as an auxiliary substance in postsurgical procedures [41]. Propolis has also attracted a substantial interest as an agent for the treatment of oral infections [46]. It has demonstrated its activity against both aerobic and anaerobic microorganisms that may be associated with caries, periodontal disease and with *Candida* infections (i.e., *Streptococcus gordonii*, *S. mutans*, *Actinomyces naeslundii*, *Fusobacterium nucleatum*, *Prevotella intermedia*, *Porphyromonas gingivalis*, *Parvimonas micra* and *Candida albicans*) [86]. Propolis extracts were able not only to retardate multispecies biofilm formation but were also active against already-formed biofilms, which contribute to the formation of plaque [86]. Detailed studies on the mechanisms of action of propolis compounds lead to the conclusion that its effect is synergistic with antibiotics [46]. A combination of propolis and ciprofloxacin resulted in their synergistic effect in the treatment of an experimental *S. aureus* keratitis. Moreover, the exposure of *Salmonella Typhi* to amoxicillin, ampicillin and cephalexin combined with propolis caused a reduction in the resistance to these antimicrobial agents, and a synergistic effect of this product with chloramphenicol, tetracycline and neomycin has been observed [90]. The wound-healing properties of propolis allowed Barud et al. [91] to demonstrate its applicability with biocellulose membranes as a wound-healing agent. Such observations allow one to assume that the combination of propolis with biocompatible agents is a promising research direction.

## 4. Conclusions

Flexible films based on sodium hyaluronate with encapsulated propolis were successfully produced. Electron microscopy allowed the shape and size of the obtained nanocapsules to be estimated (spherical nanocapsules ranging in size from 90 to 200 nm). The optimal concentration at which complete encapsulation occurs was determined, indicating at the same time that a further increase in the concentration of propolis results in partial encapsulation. The FTIR spectra confirmed the presence of propolis in the obtained films, at the same time indicating no structural changes in the components during the encapsulation processes. The DLS analysis showed that during the production of nanocapsules, there is also a reduction in the size of hyaluronic acid particles with a simultaneous increase in the stability of the system (increase in the value of the zeta potential). Polydispersity values are consistent with the results of microscopic analysis, confirming the formation of capsules, indicating that at optimal concentration they are the most homogeneous. With the increase in propolis concentration, an increase in hydrophobicity and improvement in mechanical and thermal properties of the obtained films were also observed.

The described antimicrobial properties of propolis extracts, coupled with the remarkable wound-healing properties, make this substance one of the most promising agents in the production of biomaterials used in dermatology and other branches of medicine. The combination of encapsulated propolis and its hyaluronic acid matrix, due to their biocompatibility and biodegradability, could allow one to obtain an effective material for the production of dressings to be applied on infected wounds.

## Figures and Tables

**Table 1:** Growth inhibition zones (mm) for bacterial and *Candida* strains, as a result of application of films containing three propolis concentrations.

Species	Origin	HP1	HP2	HP3	C
*M. luteus*	skin	24.00	30.00	23.75	0
*S. aureus*	skin	26.00	24.50	22.75	0
*S. cohnii*	skin	20.00	19.50	24.25	0
*S. epidermidis*	skin	24.50	27.50	26.25	0
*S. haemolyticus*	skin	24.50	23.00	22.5	0
*S. pasteuri*	skin	25.00	23.50	23.25	0
*S. saprophyticus*	skin	24.00	23.50	24.25	0
*S. warneri*	skin	24.00	22.50	28.75	0
mean	-	24.00	24.25	24.47	0
Standard deviation		1.75	3.21	2.08	
Coefficient of variation (%)		7.30	13.23	8.52	
*C. albicans* 1	type strain	21.00	21.00	25.00	0
*C. albicans 2*	anus	21.50	22.00	24.00	0
*C. albicans* 3	cheek	20.00	21.50	23.00	0
*C. albicans* 4	mouth	21.50	22.50	21.00	0
*C. formata*	mouth	24.00	24.50	26.00	0
*C. glabrata*	vagina	25.50	26.00	27.00	0
*C. guillermondii*	skin under eye	19.50	20.50	21.50	0
*C. inconspicua* 1	vagina	22.50	24.00	25.00	0
*C. inconspicua* 2	vagina	24.00	22.00	22.50	0
*C. inconspicua* 3	skin	21.20	22.90	25.10	0
*C. inconspicua* 4	mouth	17.50	19.50	20.00	0
*C. inconspicua* 5	throat	18.50	19.50	20.00	0
*C. krusei*	vagina	23.00	24.00	25.50	0
*C. parapsilosis* 1	type strain	20.00	22.50	23.00	0
*C. parapsilosis* 2	anus	22.00	23.50	25.50	0
*C. parapsilosis* 3	skin	22.50	23.00	26.50	0
*C. parapsilosis* 4	skin	20.00	23.50	27.00	0
*C. parapsilosis* 5	throat	15.00	19.50	20.00	0
mean		21.07	22.33	23.76	
Standard deviation		2.52	1.84	2.45	
Coefficient of variation (%)		11.96	8.23	10.33	

**Table 2:** Color parameters of the films.

Sample	L* (D65)	a* (D65)	b* (D65)
ControlH	91.1 ± 0.03 ^a^	−0.5 ± 0.01 ^a^	3.8 ± 0.04 ^d^
HP1	87.8 ± 0.19 ^b^	−3.7 ± 0.10 ^d^	30.4 ± 0.74 ^a^
HP2	90.5 ± 0.30 ^a^	−2.8 ± 0.07 ^c^	17.2 ± 0.34 ^b^
HP3	90.8 ± 0.45 ^a^	−1.0 ± 0.03 ^b^	11.2 ± 0.60 ^c^

The measurement was performed in 3 repetitions. The parameters in columns (value ± standard deviation) denoted with the same letters (a, b, c, d) do not differ statistically at significant level 0.05.

**Table 3:** Mechanical properties of films.

Sample	Thickness [mm]	TS [MPa]	E [%]
ControlH	0.64 ± 0.062 ^a^	44.41 ± 9.85 ^b^	4.0 ± 0.3 ^a^
HP1	0.067 ± 0.022 ^a^	43.59 ± 5.20 ^b^	4.0 ± 0.8 ^a^
HP2	0.063 ± 0.023 ^a^	44.20 ± 9.17 ^b^	3.8 ± 0.5 ^a^
HP3	0.061 ± 0.013 ^a^	57.08 ± 8.11 ^a^	4.0 ± 0.1 ^a^

TS—tensile strength; E—elongation at break. The measurement was performed in 3 repetitions. The parameters in columns (value ± standard deviation) denoted with the same letters (a, b) do not differ statistically at significant level 0.05.

**Table 4:** Wetting angles, surface free energy (SFE), zeta potential, particle size and polydispersity index (PDI).

Sample	Contact Angle [°]		Surface Free Energy [mJ/m^2^]			Zeta Potential [mV]	Particle Size [nm]	PDI
	Water	Diiodomethane	Dispersive	Polar	Total Free Energy			
ControlH	58.80 ± 2.5	42.50 ± 3.1	33.44 ± 1.3	15.67 ± 0.9	49.11 ± 2.2	−5.12 ± 0.7	13,000 ± 450	0.42
HP1	28.10 ± 2.4	41.50 ± 1.5	28.30 ± 0.6	37.41 ± 0.8	65.70 ± 1.3	−53.70 ± 2.3	8000 ± 150	0.25
HP2	46.43 ± 2.7	43.60 ± 4.1	30.03 ± 1.9	25.29 ± 0.6	55.32 ± 2.5	−69.40 ± 3.5	8300 ± 200	0.23
HP3	76.85 ± 3.1	40.70 ± 2.7	39.57 ± 0.8	4.61 ± 1.0	44.18 ± 1.8	−52.40 ± 2.7	8500 ± 180	0.27

**Table 5:** Moisture content and moisture uptake.

Sample	Moisture Content [%]	Moisture Uptake [%]
ControlH	7.87 ± 0.08 ^b^	6.94 ± 0.04 ^b^
HP1	11.11 ± 0.03 ^a^	7.35 ± 0.04 ^a^
HP2	7.41 ± 0.08 ^c^	6.67 ± 0.06 ^c^
HP3	2.02 ± 0.09 ^d^	0.48 ± 0.04 ^d^

The measurement was performed in 3 repetitions. The parameters in columns (value ± standard deviation) denoted with the same letters (a, b, c, d) do not differ statistically at significant level 0.05.

**Table 6:** Analysis of differential scanning calorimetry of analysis of the films with propolis.

	Endothermic Process				
	T_o_ (°C)	T_p_ (°C)	T_e_ (°C)	ΔH (J/g)	T_g_ (°C)
ControlH	165.6 ± 6.9	167.4 ± 7.2	171.3 ± 7.0	188.7 ± 7.4	166.5
HP1	169.0 ± 8.9	171.6 ± 8.4	176.6 ± 9.0	154.7 ± 11.6	170.3
HP2	181.4 ± 9.3	183.7 ± 9.5	188.2 ± 10.0	147.3 ± 0.9	182.6
HP3	183.3 ± 6.3	185.4 ± 5.7	190.0 ± 5.7	148.2 ± 4.4	184.3
	**Exothermic Process**				
	**T_o_ (** **°** **C)**	**T_p_ (** **°** **C)**	**T_e_ (** **°** **C)**	**ΔH (** **J/g** **)**	**T_g_ (°C)**
ControlH	228.1 ± 2.0	233.8 ± 0.8	241.9 ± 1.5	−220.5 ± 11.2	230.9
HP1	217.7 ± 2.1	227.3 ± 0.8	236.9 ± 0.8	−197.6 ± 3.1	222.5
HP2	216.0 ± 0.7	226.5 ± 1.1	237.1 ± 0.2	−197.1 ± 7.73	221.2
HP3	221.7 ± 1.5	229.0 ± 1.1	238.1 ± 2.5	−202.0 ± 7.77	225.4

The parameters in columns: value ± standard deviation.

## Data Availability

The data presented in this study are available upon request from the corresponding author.
